# Supplementary material for: Diurnal oscillations of MRI metrics in the brains of male participants
Source: Nat Commun. 2023 Nov 3;14:7044. doi: 10.1038/s41467-023-42588-6 (PMC10624685; doi:10.1038/s41467-023-42588-6)
Supplement: Supplementary file 1 — Supplementary Information [file 41467_2023_42588_MOESM1_ESM.pdf]

Diurnal oscillations of MRI metrics in the brains of male participants

Supplementary Information

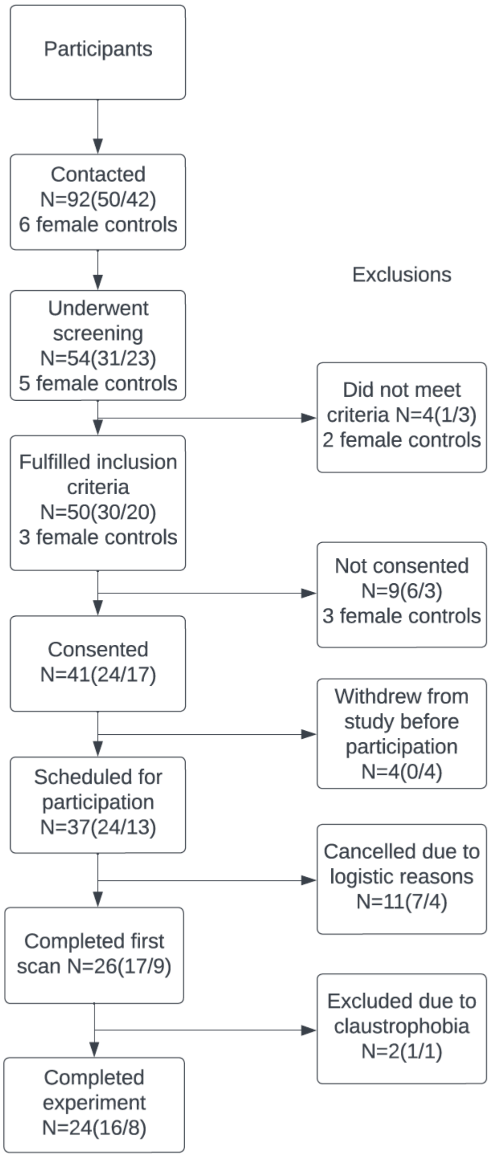

Supplementary Fig. 1 | Recruitment and retention flowchart.
Numbers (n/n) in the parentheses are control participants (first) and participants with BPD (second).

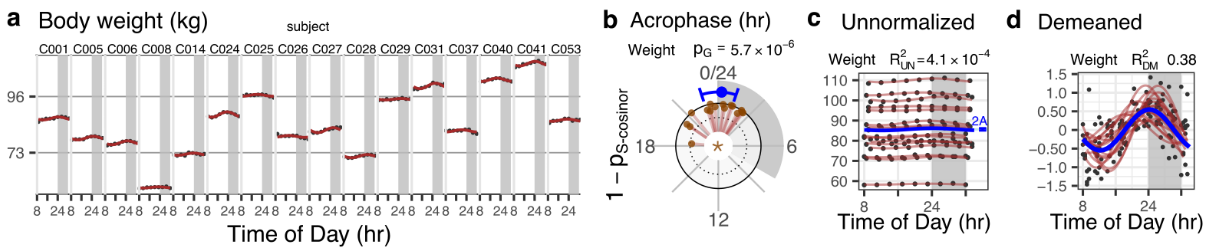

Supplementary Fig. 2 | Diurnal oscillations in body weight of control subjects. Identical methods as in Figure 2 were applied to body weight data. **a** Body weight (y-axis) over a 24-hr period (x-axis) for each subject (columns). S-cosinor fit lines are shown with residuals connecting dots to the fit. **b** Circular plots of S- and G-cosinor acrophases. Rotational axis: time of day (0-24hrs). Brown dots: S-cosinor acrophase for each subject. Radial axis: inverse S-cosinor one-tailed F-test uncorrected p-values ($1\text{-}p_{S\text{-cosinor}}$) ranging from zero to one. Blue lines: significant ($p_G<0.05$) G-cosinor acrophase estimates with corresponding 95% confidence interval. Plot panel title indicates the G-cosinor one-tailed F-test uncorrected p-value (p_G). Panel centre: Star indicates the one-tailed chi-square acrophase-agnostic test was significant ($p<0.05$). **c** Body weight as in (a), with S-cosinor fit lines in brown; G-cosinor curve shown in blue where the blue bracket and shading indicate peak-to-peak amplitude (2*A) of the G-cosinor model. Plot panel title indicates the proportion of variance explained by the G-cosinor model (R^2_{UN}). **d** Within-subject demeaned values (black dots) reveal oscillatory effects of the G-cosinor model, represented as a thick blue line. Brown lines: Individual S-cosinor fits. Plot panel title indicates the proportion of variance explained by the G-cosinor model on the demeaned data (R^2_{DM}).

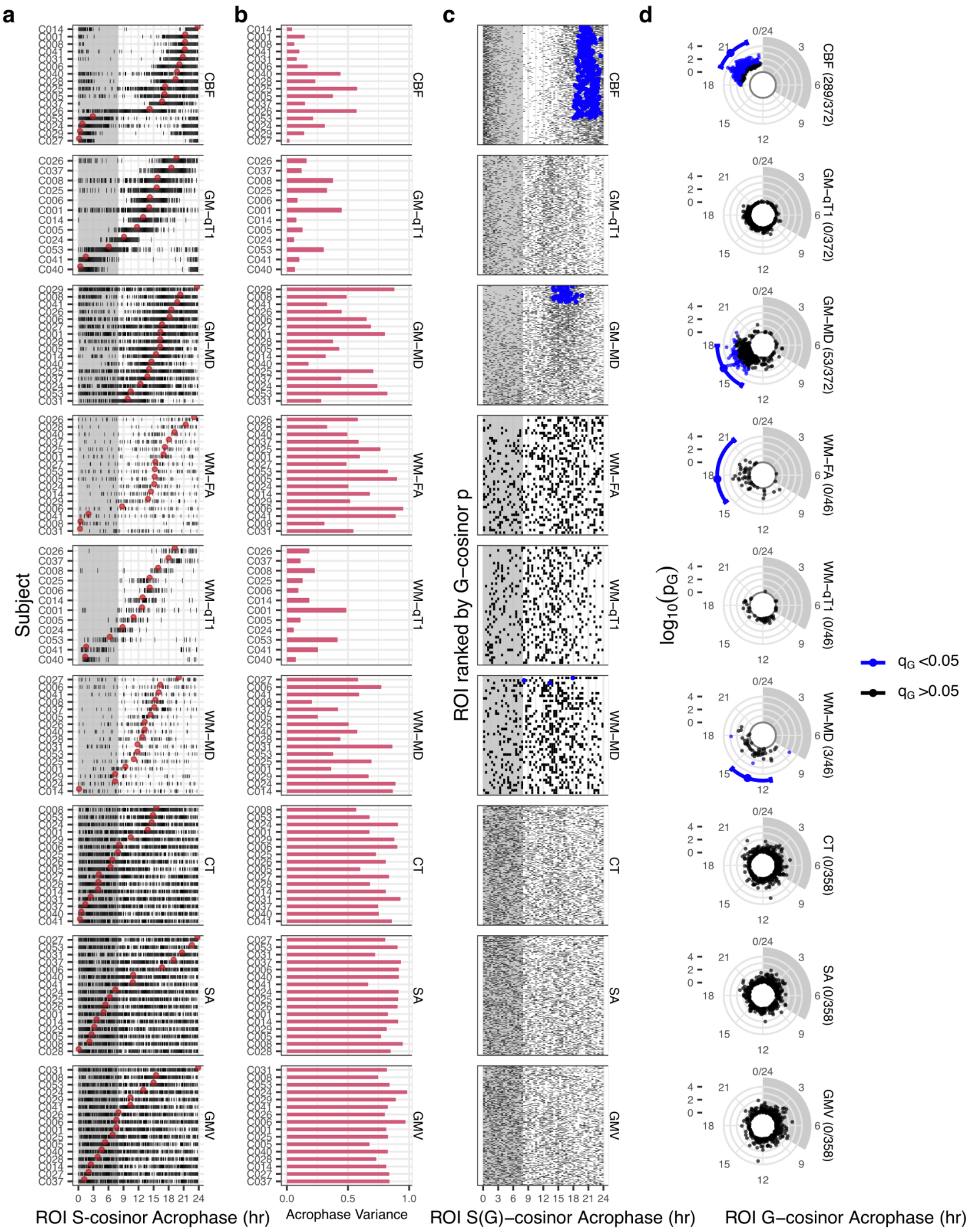

Supplementary Fig. 3 | Regional cosinor statistics for all metrics.

a-b As described in Figure 3a. **a** Subject-level cosinor (S-cosinor) acrophase estimates are plotted as vertical ticks for each ROI (x-axis), individually for each subject (y-axis). For each metric, subjects are sorted according to their whole brain subject-level acrophase (brown dots). Blue tick: significant whole-brain group-level cosinor (G-cosinor) acrophase estimate. **b** Red bars indicate each subject's acrophase variance across all ROIs. **c** For each ROI (y-axis - sorted by ROI G-cosinor p-value within each metric, p_G), each subject's cosinor acrophase estimate (x-axis) is shown as a black tick to illustrate within-ROI consistency. ROIs with G-cosinor significant acrophases (FDR $q<0.05$) are shown as blue dots. **d** As described in Figure 3c. Blue dots: estimates of the G-cosinor acrophase for each ROI shown on the rotational axis in polar coordinates where the radial axis is the negative logarithm of the G-cosinor p-value (p_G). Blue intervals: significant whole brain acrophases and 95% confidence interval. Panel centres: the number of significant ROIs to total ROIs. CBF: cerebral blood flow; GMV: grey matter volume; CT: cortical thickness: GM-MD: grey matter mean diffusivity; GM-qT1: grey matter quantitative T1 relaxation time; SA: surface area; WM-FA white matter skeleton fractional anisotropy; WM-MD: white matter skeleton mean diffusivity; WM-qT1: white matter skeleton quantitative T1 relaxation time; ROI: region of interest. Source data are provided as a Source Data file.

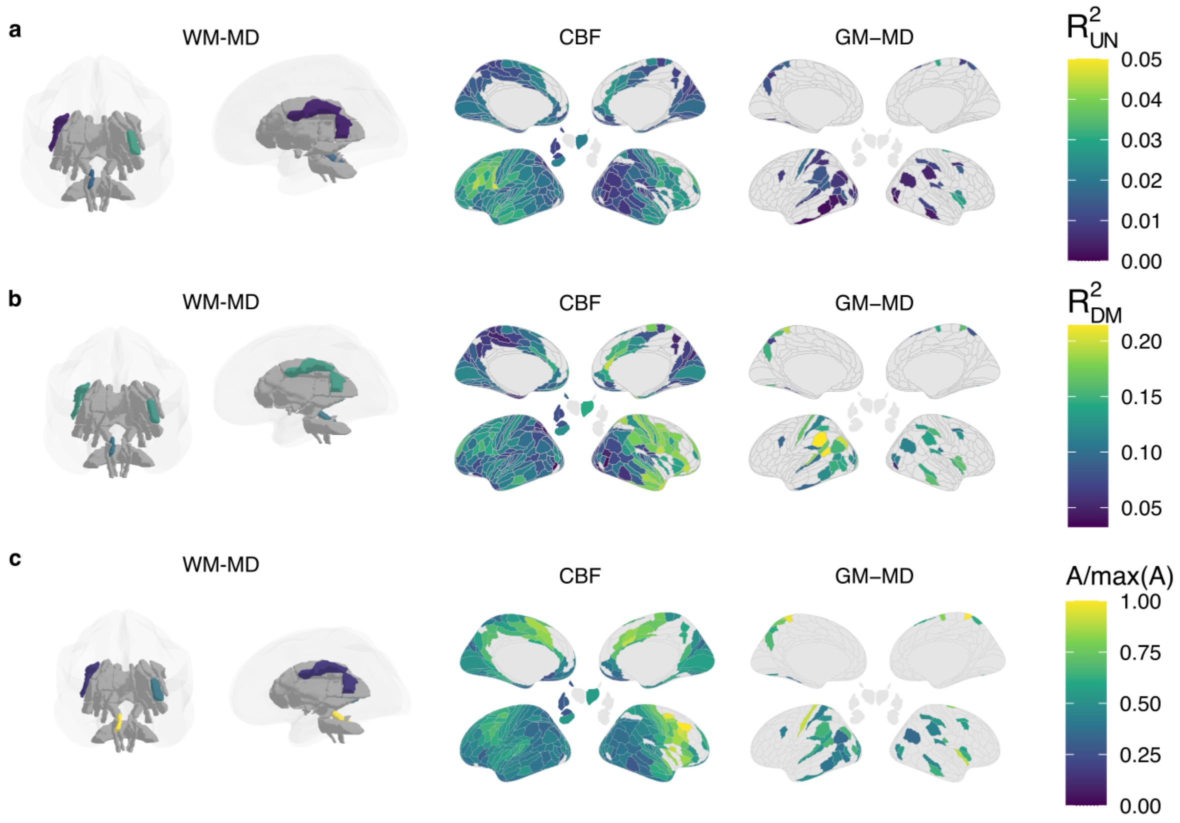

Supplementary Fig. 4 | Regional G-cosinor statistics in controls.
Spatial distribution of G-cosinor statistics for CBF, WM- and GM-MD regions with G-cosinor FDR $q<0.05$ from the one-tailed F-test. **a** Unnormalized proportion of variance explained, R^2_{UN}, **b** Normalized (demeaned) proportion of variance explained, R^2_{DM} and **c** Amplitudes (A), scaled relative to the maximum amplitude within metric for visualization purposes. CBF: cerebral blood flow; WM-MD: white matter skeleton mean diffusivity; GM-MD: cortical grey matter mean diffusivity. Source data are provided as a Source Data file.

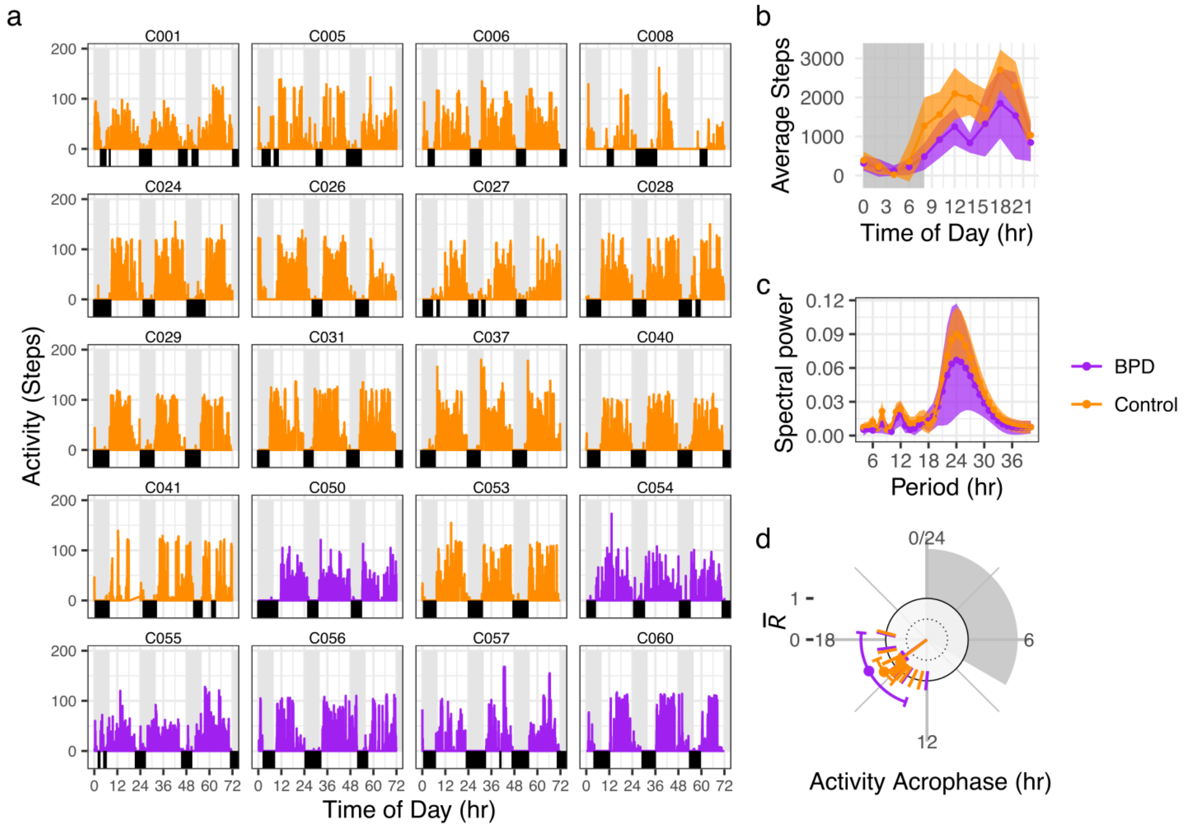

Supplementary Fig. 5 | Actigraphy in units of steps registered.

a 3 days of raw step data from actigraphy with sleep (black bars). **b** Aggregated step data over 2 hour bins dotted lines: within group means; shaded area: 95% confidence interval of the mean (confidence band). **c** Spectral power of periods (R^2 of S-cosinor fit) ranging from 4hr to 40hr on step data for each subject averaged within group with mean and 95% confidence interval shown as a confidence band. **d** Acrophases of actigraphy step data oscillations (G-cosinor $p<0.05$) and 95% confidence intervals shown on outer orbit. S-cosinor acrophases for each subject are shown as ticks. Each arrow points in the direction of the mean acrophase across control subjects (orange arrow), and participants with BPD patients (purple arrow) and its length represents a measure of acrophase consistency across subjects (1 − acrophase variance). BPD: participants with bipolar disorder. Source data are provided as a Source Data file.

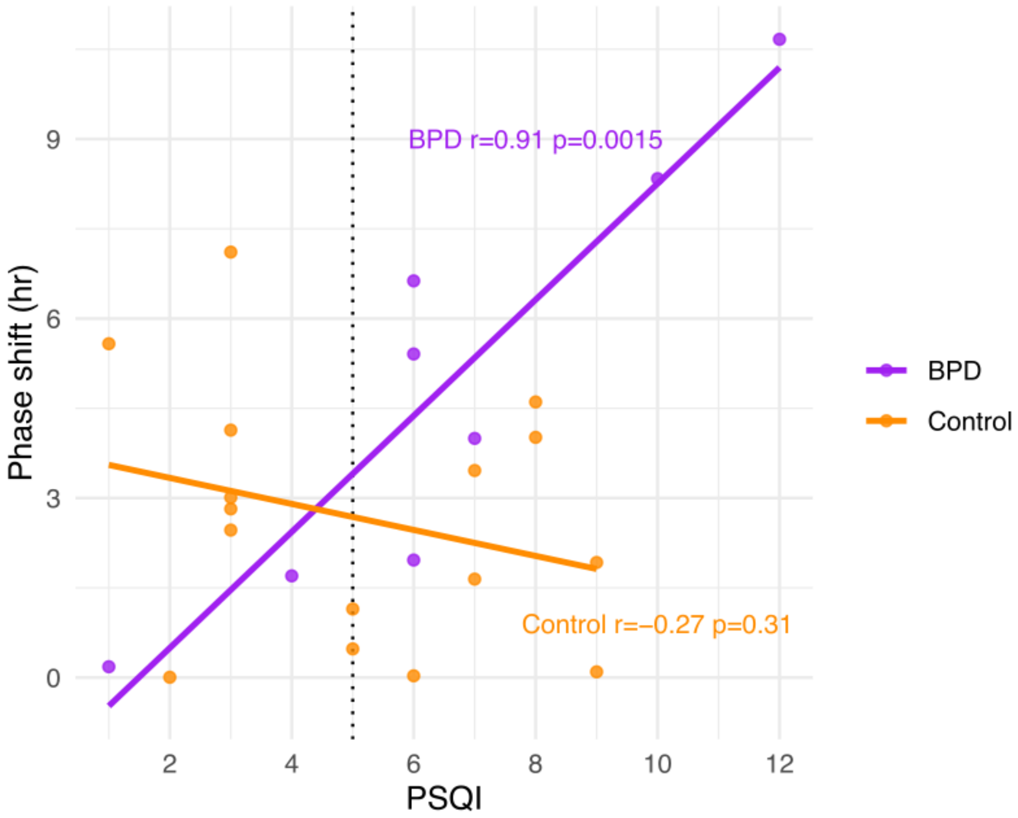

Supplementary Fig. 6 | CBF phase deviation correlates with subjective sleep quality in BPD. Phase shift was calculated for each subject as the minimum arc length relative to the G-cosinor acrophase in the combined group. Sleep quality was measured with the PSQI, where higher values indicate poor sleep quality. The dotted vertical line indicates the PSQI threshold for good (<5) and poor (>5) sleep quality[1]. Each group (n=16,8 for control and BPD groups, respectively) was tested with Pearson's correlation (results in panel). BPD: participants with bipolar disorder; PSQI: Pittsburgh Sleep Quality Index. Orange dots and lines: controls; Purple dots and lines: participants with bipolar disorder.

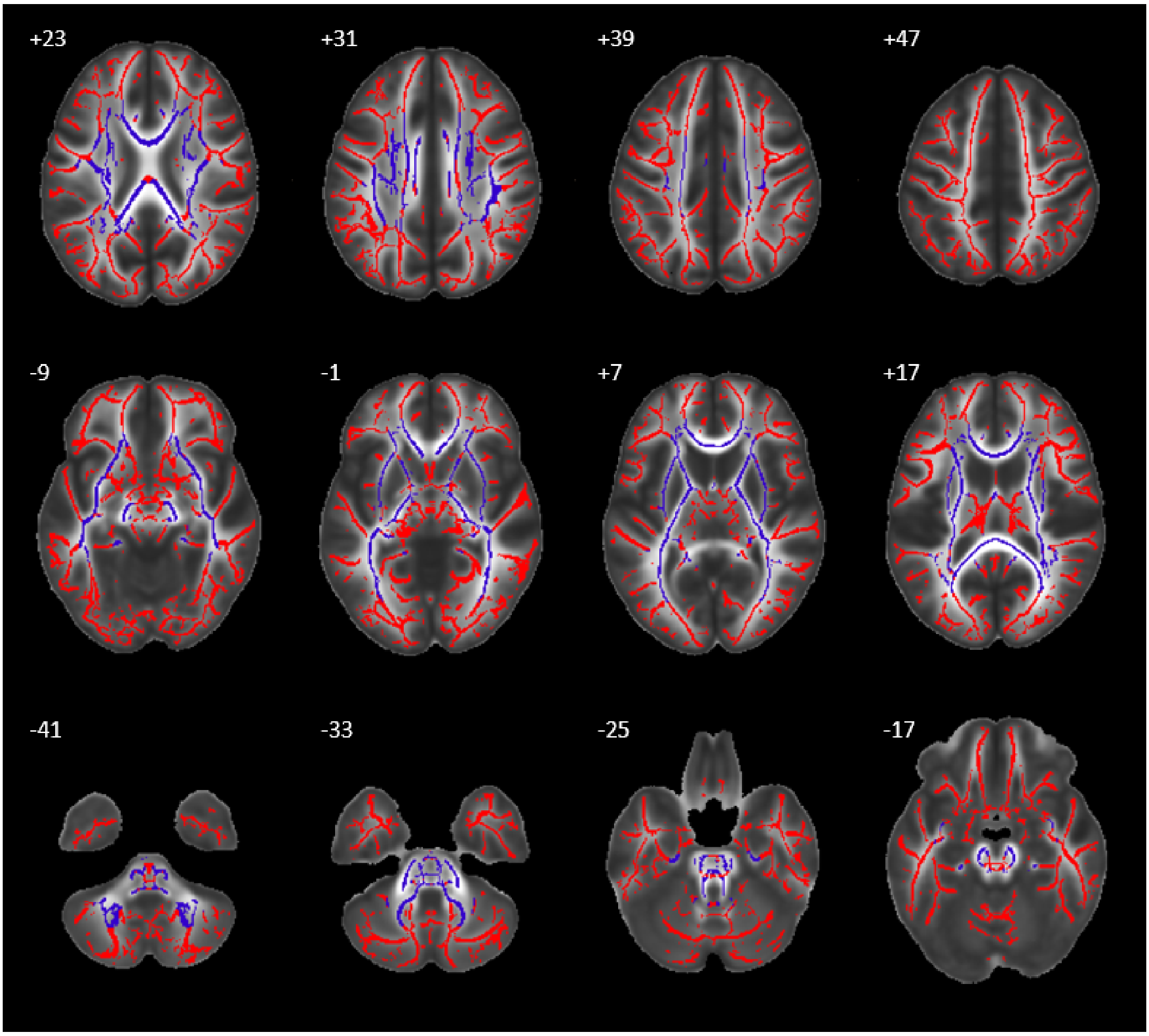

Supplementary Fig. 7 | TBSS skeleton overlaid on the mean FA map.
Blue: voxels included in the regional analyses, obtained from the JHU Atlas[2]. Red: additional voxels included in the whole-skeleton analysis. Slice locations are indicated at the upper left of each slice, left is on the left, and up is anterior. TBSS: tract-based spatial statistics, FA: fractional anisotropy.

Metric	Subject	Explained sum of squares	Mean sum of squares	$F_{8,18}$	p
CBF	C028	1.40E+02	1.80E+01	22.3	7.80E-08
CBF	C031	3.80E+02	4.70E+01	17.8	4.50E-07
GM-MD	C027	4.80E-05	6.00E-06	21.9	9.00E-08
GM-MD	C029	2.00E-05	2.50E-06	19.3	2.40E-07
WM-FA	C027	2.40E-04	3.00E-05	5.8	9.40E-04
WM-FA	C029	2.70E-04	3.40E-05	12.5	6.50E-06
WM-MD	C027	4.90E-06	6.10E-07	19	2.70E-07
WM-MD	C029	3.80E-06	4.70E-07	11.3	1.30E-05

Supplementary Table 1 | ANOVA on whole-brain-averaged back-to-back repeated scan data. Diffusion tensor imaging or arterial spin labeling measurements were repeated three times at each of the nine time points in two subjects. A one-way ANOVA on whole-brain data assessing within- vs. between-time point variation was performed to determine if scan-to-scan variation was smaller than across-time variation. CBF: cerebral blood flow; GM-MD: grey matter mean diffusivity; WM-FA: white matter skeleton fractional anisotropy; WM-MD: white matter skeleton mean diffusivity.

Group	Metric	Oscillation parameters			G-cosinor zero amplitude			Effect size		Acrophase-agnostic test		
	Metric	MESOR [CI]	Amplitude [CI]	Acrophase [CI]	$F_{2,df}$	*df*	*p*	R^2_{UN}	R^2_{DM}	χ^2	*p*	Units
Controls	**CBF**	**63 [51,76]**	**4.5 [2.0,7.0]**	**21 [20,23]**	**6.8**	**14**	**0.0088**	**0.020**	**0.15**	**54**	**0.0083**	**ml/g/min**
	GM-qT1	1,347 [1,316,1,377]	6.1 [0,26]	16	0.20	10	0.79	0.0050	0.0075	46	0.0042	ms
	**GM-MD**	**0.089 [0.087,0.091]**	**4.4e-04 [2.3e-04,6.5e-04]**	**16 [14,18]**	**9.5**	**14**	**0.0025**	**0.0055**	**0.20**	**65**	**0.00038**	**0.1 mm^2/s**
	**WM-FA**	**0.45 [0.44,0.45]**	**9.9e-04 [3.8e-04,0.0016]**	**18 [16,21]**	**8.8**	**14**	**0.0034**	**0.0034**	**0.084**	**48**	**0.030**	**1**
	WM-qT1	933 [910,956]	3.9 [0,19]	14	0.20	10	0.85	0.0040	0.0070	37	0.042	ms
	**WM-MD**	**0.075 [0.074,0.076]**	**2.4e-04 [1.3e-04,3.5e-04]**	**13 [11,15]**	**10.5**	**14**	**0.0017**	**0.0098**	**0.19**	**59**	**0.0023**	**0.1 mm^2/s**
	CT	2.4 [2.4,2.5]	0.0041 [0,0.0092]	6	1.8	14	0.20	0.0025	0.026	36	0.26	mm
	SA	189 [182,197]	0.20 [0,0.42]	4	2.1	14	0.16	3.8e-05	0.018	25	0.78	10^3 mm^2
	GMV	523 [499,547]	1.6 [0.32,3.0]	6	3.6	14	0.053	7.1e-04	0.069	42	0.094	10^3 mm^3
	**Weight**	**86 [79,93]**	**0.55 [0.38,0.72]**	**0 [23,1]**	**32.3**	**14**	**5.7e-06**	**4.1e-04**	**0.38**	**112**	**6.7e-11**	**kg**
BPD	CBF	50 [48,52]	1.1 [0,4.0]	0	0.30	6	0.74	0.019	0.021	23	0.11	ml/g/min
	GM-qT1	1,395 [1,338,1,451]	14 [0,44]	3	0.60	6	0.59	0.010	0.022	26	0.054	ms
	**GM-MD**	**0.092 [0.089,0.094]**	**4.8e-04 [2.0e-04,7.6e-04]**	**15 [12,19]**	**6.9**	**6**	**0.027**	**0.0094**	**0.15**	**31**	**0.013**	**0.1 mm^2/s**
	**WM-FA**	**0.45 [0.44,0.45]**	**0.0019 [0.0012,0.0025]**	**17 [15,19]**	**21.3**	**6**	**0.0019**	**0.015**	**0.23**	**29**	**0.021**	**1**
	WM-qT1	968 [934,1,001]	10 [0,29]	3	1.0	6	0.44	0.012	0.021	24	0.078	ms
	WM-MD	0.075 [0.074,0.076]	1.7e-04 [2.2e-05,3.1e-04]	13	4.2	6	0.074	0.0043	0.15	33	0.0057	0.1 mm^2/s
	CT	2.4 [2.4,2.5]	0.0028 [0,0.0081]	15	0.70	6	0.51	0.0011	0.0099	18	0.29	mm
	**SA**	**185 [171,198]**	**0.52 [0.20,0.84]**	**3 [1,7]**	**6.7**	**6**	**0.030**	**8.1e-04**	**0.14**	**19**	**0.27**	10^3 mm^2
	GMV	510 [475,545]	0.77 [0,2.2]	4	0.70	6	0.54	3.6e-04	0.017	18	0.31	10^3 mm^3
	**Weight**	**88 [77,99]**	**0.61 [0.21,1.0]**	**24 [23,2]**	**6.2**	**6**	**0.034**	**0.0046**	**0.37**	**42**	**0.00036**	**kg**
Combined	**CBF**	**59 [50,67]**	**3.2 [1.3,5.2]**	**21 [20,23]**	**5.9**	**22**	**0.0090**	**0.013**	**0.10**	**77**	**0.0046**	**ml/g/min**
	GM-qT1	1,366 [1,338,1,394]	2.7 [0,19]	1	0.10	18	0.95	5e-04	0.0013	72	0.0013	ms
	**GM-MD**	**0.090 [0.088,0.091]**	**4.5e-04 [3e-04,6.1e-04]**	**16 [14,17]**	**17.5**	**22**	**2.9e-05**	**0.0049**	**0.18**	**97**	**3.7e-05**	**0.1 mm^2/s**
	**WM-FA**	**0.45 [0.44,0.45]**	**0.0013 [8e-04,0.0018]**	**17 [16,19]**	**19.2**	**22**	**1.5e-05**	**0.0059**	**0.13**	**78**	**0.0039**	**1**
	WM-qT1	947 [928,966]	1.9 [0,13]	4	0.10	18	0.94	4.4e-04	0.0011	61	0.015	ms
	**WM-MD**	**0.075 [0.075,0.076]**	**2.1e-04 [1.3e-04,2.9e-04]**	**13 [12,15]**	**13.9**	**22**	**0.00012**	**0.0084**	**0.18**	**93**	**9.9e-05**	**0.1 mm^2/s**
	CT	2.4 [2.4,2.5]	0.0023 [0,0.0062]	8	0.70	22	0.52	9.7e-04	0.0087	55	0.22	mm
	**SA**	**188 [182,194]**	**0.31 [0.13,0.49]**	**4 [1,7]**	**6.7**	**22**	**0.0053**	**1.7e-04**	**0.043**	**44**	**0.61**	**10^3 mm^2**
	**GMV**	**519 [500,537]**	**1.3 [0.34,2.3]**	**6 [3,9]**	**3.8**	**22**	**0.038**	**5.4e-04**	**0.048**	**61**	**0.096**	**10^3 mm^3**
	**Weight**	**86 [81,92]**	**0.57 [0.42,0.72]**	**0 [23,1]**	**29.4**	**22**	**6.1e-07**	**0.0018**	**0.38**	**154**	**3.6e-13**	**kg**

Supplementary Table 2 | Cosinor statistics for all MRI metrics at the whole-brain level.
When p >0.05, lower bounds of amplitude 95% CI are shown as "0" and acrophase 95% CI is not shown. Rows with G-cosinor p<0.05 are in bold. F-tests and chi-square tests are one-tailed, all p-values are not corrected for multiple testing. MESOR: midline estimating statistic of rhythm; CI: confidence interval; df: degrees of freedom; CBF: cerebral blood flow; GM-qT1: grey matter quantitative T1 relaxation time; GM-MD: grey matter mean diffusivity; WM-FA white matter skeleton fractional anisotropy; WM-qT1: white matter skeleton quantitative T1 relaxation time; WM-MD: white matter skeleton mean diffusivity; CT: cortical thickness: SA: surface area; GMV: grey matter volume; R^2_{UN}: unnormalized proportion of variance explained; R^2_{DM}: normalized (demeaned) proportion of variance explained; BPD: participants with bipolar disorder.

	Oscillation parameters			*G-cosinor zero amplitude*		
Metric	MESOR [CI]	Amplitude [CI]	Acrophase [CI]	$F_{2,df}$	*df*	*p*
**CBF**	**63 [51,76]**	**3.8 [1.0,6.5]**	**19 [16,21]**	**4.4**	**14**	**0.033**
GM-qT1	1,347 [1,317,1,377]	3.2 [0,29]	20	0.034	10	0.97
**GM-MD**	**0.089 [0.087,0.091]**	**4.5e-04 [2e-04,6.9e-04]**	**16 [14,18]**	**7.2**	**14**	**0.0072**
**WM-FA**	**0.45 [0.44,0.45]**	**0.0012 [3.5e-04,0.0021]**	**20 [16,23]**	**4.3**	**14**	**0.035**
WM-qT1	933 [910,957]	1.6 [0,21]	21	0.015	10	0.99
**WM-MD**	**0.075 [0.074,0.076]**	**2.9e-04 [1.5e-04,4.2e-04]**	**14 [12,16]**	**10**	**14**	**0.0017**
CT	2.4 [2.4,2.5]	0.0032 [0,0.012]	4	0.31	14	0.74
SA	189 [182,197]	0.31 [0,0.88]	12	0.62	14	0.55
GMV	523 [499,547]	1.2 [0,2.7]	7.3	1.6	14	0.25

Supplementary Table 3 | Whole-brain G-cosinor models in controls after covarying for body weight.

Each participant first had an S-cosinor model fit with their weight prior to each scanning session as an additional covariate. Amplitudes and acrophases of these fits were used for the one-tailed G-cosinor F-test without correction for multiple testing. Rows with $p<0.05$ are in bold. MESOR: midline estimating statistic of rhythm; df: degrees of freedom; CI: confidence interval; CBF: cerebral blood flow; GM-qT1: grey matter quantitative T1 relaxation time; GM-MD: grey matter mean diffusivity; WM-FA white matter skeleton fractional anisotropy; WM-qT1: white matter skeleton quantitative T1 relaxation time; WM-MD: white matter skeleton mean diffusivity; CT: cortical thickness: SA: surface area; GMV: grey matter volume;

		Oscillation parameters			*G-cosinor zero amplitude*	*Effect size*		*Acrophase-agnostic test*
	Metric	MESOR	Amplitude	Acrophase	% p<0.05 (#/total; # q<0.05)	R^2_{UN}	R^2_{DM}	%p<0.05 (#/total; # q<0.05)
Control	CBF	46-82	1.8-9.2	18-23	86% (319/372; 289)	0.0062-0.047	0.033-0.21	59% (218/372; 128)
	GM-MD	0.073-0.10	4.5e-04-0.0013	14-20	38% (141/372; 53)	5.9e-04-0.030	0.052-0.21	39% (144/372; 36)
	WM-MD	0.071-0.085	2.1e-04-0.0013	8-18	26% (12/46; 3)	0.0042-0.028	0.096-0.13	17% (8/46; 1)
	WM-FA	—	—	—	26% (12/46; 0)	—	—	13% (6/46; 0)
	CT	—	—	—	7.5% (27/358; 0)	—	—	7.8% (28/358; 0)
	GMV	—	—	—	7.5% (27/358; 0)	—	—	5.6% (20/358; 0)
	SA	—	—	—	5.9% (21/358; 0)	—	—	5.3% (19/358; 0)
	GM-qT1	—	—	—	0% (0/372; 0)	—	—	41% (151/372; 4)
	WM-qT1	—	—	—	0% (0/46; 0)	—	—	8.7% (4/46; 0)
BPD	WM-FA	0.51-0.67	0.0028-0.0046	15-20	22% (10/46; 5)	0.0060-0.035	0.084-0.25	11% (5/46; 0)
	CT	2.5	0.062	4	3.6% (13/358; 1)	0.096	0.19	7.0% (25/358; 0)
	GM-MD	—	—	—	20% (75/372; 0)	—	—	28% (103/372; 16)
	WM-MD	—	—	—	11% (5/46; 0)	—	—	17% (8/46; 0)
	GMV	—	—	—	4.7% (17/358; 0)	—	—	5.3% (19/358; 0)
	SA	—	—	—	4.2% (15/358; 0)	—	—	5.3% (19/358; 0)
	GM-qT1	—	—	—	0.81% (3/372; 0)	—	—	23% (85/372; 0)
	CBF	—	—	—	0% (0/372; 0)	—	—	27% (99/372; 7)
	WM-qT1	—	—	—	0% (0/46; 0)	—	—	17% (8/46; 0)
Combined	CBF	42-72	1.7-6.1	18-24	83% (310/372; 265)	0.0045-0.034	0.028-0.18	75% (280/372; 246)
	GM-MD	0.072-0.11	2.7e-04-0.0015	8-19	55% (206/372; 168)	0-0.024	0.024-0.18	51% (188/372; 136)
	WM-FA	0.49-0.66	0.0021-0.0029	13-18	35% (16/46; 8)	0.0038-0.013	0.046-0.11	6.5% (3/46; 0)
	WM-MD	—	—	—	33% (15/46; 0)	—	—	28% (13/46; 3)
	GMV	—	—	—	7.0% (25/358; 0)	—	—	6.7% (24/358; 0)
	SA	—	—	—	6.4% (23/358; 0)	—	—	8.1% (29/358; 0)
	CT	—	—	—	3.6% (13/358; 0)	—	—	6.4% (23/358; 0)
	GM-qT1	—	—	—	0% (0/372; 0)	—	—	59% (218/372; 97)
	WM-qT1	—	—	—	0% (0/46; 0)	—	—	8.7% (4/46; 0)

Supplementary Table 4 | Summary of cosinor statistics for all MRI metrics at the regional level. Ranges are given for all ROIs within each metric with G-cosinor FDR rate q<0.05. p-values are not corrected for multiple testing. MESOR: midline estimating statistic of rhythm; CBF: cerebral blood flow; GM-qT1: grey matter quantitative T1 relaxation time; GM-MD: grey matter mean diffusivity; WM-FA white matter skeleton fractional anisotropy; WM-qT1: white matter skeleton quantitative T1 relaxation time; WM-MD: white matter skeleton mean diffusivity; CT: cortical thickness: SA: surface area; GMV: grey matter volume; R^2_{UN}: unnormalized proportion of variance explained; R^2_{DM}: normalized (demeaned) proportion of variance explained; BPD: participants with bipolar disorder.

Supplementary References

1. Buysse, D. J., Reynolds, C. F., 3rd, Monk, T. H., Berman, S. R. & Kupfer, D. J. The Pittsburgh Sleep Quality Index: a new instrument for psychiatric practice and research. *Psychiatry Res.* **28**, 193–213 (1989).
2. Hua, K. *et al.* Tract probability maps in stereotaxic spaces: analyses of white matter anatomy and tract-specific quantification. *Neuroimage* **39**, 336–347 (2008).
