# Supplementary material for: Diurnal oscillations of MRI metrics in the brains of male participants
Source: Nat Commun. 2023 Nov 3;14:7044. doi: 10.1038/s41467-023-42588-6 (PMC10624685; doi:10.1038/s41467-023-42588-6)
Supplement: Supplementary file 2 — Reporting Summary [file 41467_2023_42588_MOESM2_ESM.pdf]

nature portfolio

Corresponding author(s): Art Petronis
Last updated by author(s): Sep 19, 2023

Reporting Summary

Nature Portfolio wishes to improve the reproducibility of the work that we publish. This form provides structure for consistency and transparency in reporting. For further information on Nature Portfolio policies, see our Editorial Policies and the Editorial Policy Checklist.

Statistics

For all statistical analyses, confirm that the following items are present in the figure legend, table legend, main text, or Methods section.

n/a	Confirmed	
☐	☒	The exact sample size (n) for each experimental group/condition, given as a discrete number and unit of measurement
☐	☒	A statement on whether measurements were taken from distinct samples or whether the same sample was measured repeatedly
☐	☒	The statistical test(s) used AND whether they are one- or two-sided<br>*Only common tests should be described solely by name; describe more complex techniques in the Methods section.*
☐	☒	A description of all covariates tested
☐	☒	A description of any assumptions or corrections, such as tests of normality and adjustment for multiple comparisons
☐	☒	A full description of the statistical parameters including central tendency (e.g. means) or other basic estimates (e.g. regression coefficient) AND variation (e.g. standard deviation) or associated estimates of uncertainty (e.g. confidence intervals)
☐	☒	For null hypothesis testing, the test statistic (e.g. F, t, r) with confidence intervals, effect sizes, degrees of freedom and P value noted<br>*Give P values as exact values whenever suitable.*
☒	☐	For Bayesian analysis, information on the choice of priors and Markov chain Monte Carlo settings
☐	☒	For hierarchical and complex designs, identification of the appropriate level for tests and full reporting of outcomes
☐	☒	Estimates of effect sizes (e.g. Cohen's d, Pearson's r), indicating how they were calculated

Our web collection on statistics for biologists contains articles on many of the points above.

Software and code

Policy information about availability of computer code

Data collection	MRI data were acquired using a 3.0-Tesla GE Discovery MR750, General Electric Medical Systems, Milwaukee, WI, USA. Software version: DV26.0_R02_1810.b
Data analysis	T1-weighted image processing and cortical labeling was accomplished using Freesurfer (version 7.1.1, http://surfer.nmr.mgh.harvard.edu/fswiki) and Advanced Normalization Tools (ANTs) version 2.3.3 (http://stnava.github.io/ANTs). N4 bias correction was used as implemented in ANTs. Tools used from Freesurfer included: mri_robust_template, the surface-based stream, and the volume-based subcortical stream. Tools used from ANTs included: antsBrainExtraction.sh with the OASIS-30 Atropos template41 (https://mindboggle.info/data.html). ANTs tools were used to calculate transformation matrices to T1-weighted images for diffusion-weighted images, the scanner-provided cerebral blood flow maps and T1 relaxation maps. Regions of interest were extracted using the Human Connectome Project Multi-Modal Parcellation atlas (version 1, HCP_MMP1, MMP1.0 210V, https://balsa.wustl.edu/976l8) via multiAltasTT (https://github.com/faskowit/multiAtlasTT.git), Freesurfer , and the Johns Hopkins University white matter atlases provided with the FSL software (https://fsl.fmrib.ox.ac.uk/fsl/fslwiki/Atlases).<br>Diffusion-weighted data were processed with FSL (version 6.0.3, https://fsl.fmrib.ox.ac.uk/fsl/fslwiki/FSL) and the topup, eddy and dtifit tools. The white matter skeleton was calculated using FSL's tract-based spatial statistics tool (TBSS, https://fsl.fmrib.ox.ac.uk/fsl/fslwiki/TBSS) and a modified version of FSL's registration procedures that implemented ANTs linear and non-linear transformations prior to skeletonization (build e934eb2, https://github.com/trislett/ants_tbss).<br>The T1mapping pipeline used the halfway_flirt command from FSL's SIENA pipeline (https://fsl.fmrib.ox.ac.uk/fsl/fslwiki/SIENA) and FSL's Brain Extraction Tool (bet, https://fsl.fmrib.ox.ac.uk/fsl/fslwiki/BET). B1 maps were generated using the two high flip angle scans via the method of slopes, and qT1 maps were computed using the variable flip angle method with a B1 correction and calibration procedure, as per the referenced papers.<br>Cerebral blood flow (CBF) maps were calculated using scanner software.

Custom code written in R ver. 4.1.3. was used to apply the referenced statistical methods to the data and to visualize the data , and is available at: https://github.com/matthewcarlucci/DiurnalMRI.

For manuscripts utilizing custom algorithms or software that are central to the research but not yet described in published literature, software must be made available to editors and reviewers. We strongly encourage code deposition in a community repository (e.g. GitHub). See the Nature Portfolio guidelines for submitting code & software for further information.

Data

Policy information about availability of data

All manuscripts must include a data availability statement. This statement should provide the following information, where applicable:

- Accession codes, unique identifiers, or web links for publicly available datasets
- A description of any restrictions on data availability
- For clinical datasets or third party data, please ensure that the statement adheres to our policy

All processed data generated in this study are available as a dataset on the Zenodo platform (https://doi.org/10.5281/zenodo.8360149). This dataset contains subject-level: processed whole-brain and ROI MRI data, body-weight data, and processed actigraphy data. Anonymized subject-level data are available to other investigators under restricted access in compliance with institutional ethics and privacy policies. Access requests can be submitted via Zenodo. The raw MRI data are protected and are not available due to institutional ethics and privacy restrictions. Source Data are provided with this paper where privacy and consent restrictions allow.

Human research participants

Policy information about studies involving human research participants and Sex and Gender in Research.

Reporting on sex and gender	Due to constraints related to the COVID shutdown of the imaging centre, all data collected were from participants self-identifying as male. This is acknowledged as a limitation in the paper.
Population characteristics	Demographic data collected are listed in Table 1.
Recruitment	Controls: Participants were recruited via word-of-mouth referrals, existing study registries and advertisements.<br>Patients: Participants with Bipolar Disorder were recruited from existing patient registries and databases at CAMH.<br><br>A single self-selection bias that we identified was (un)willingness to spend 24 hrs in the imaging facility for multiple MRI scans. We believe this did not have any impact on the results.
Ethics oversight	The CAMH Research Ethics Board approved the study. All participants provided written informed consent prior to commencement of the study, and in accordance with the Declaration of Helsinki.

Note that full information on the approval of the study protocol must also be provided in the manuscript.

Field-specific reporting

Please select the one below that is the best fit for your research. If you are not sure, read the appropriate sections before making your selection.

☒ Life sciences ☐ Behavioural & social sciences ☐ Ecological, evolutionary & environmental sciences

For a reference copy of the document with all sections, see nature.com/documents/nr-reporting-summary-flat.pdf

Life sciences study design

All studies must disclose on these points even when the disclosure is negative.

Sample size	No sample size calculations were performed. We acquired data from as many participants as possible prior to the COVID shutdown of the research facility.
Data exclusions	MRI technical issues were identified for participant C063's data for session 2, so their data were fit with 8 timepoints (p.18, document).<br>Participant C056's data for two of the four the T1 relaxation mapping acquisitions were not usable due patient motion for session 9, so their qT1 data were fit with 8 timepoints (p18, document).
Replication	This study was designed to identify whether diurnal oscillations were present, not as a replication study.
Randomization	No randomization was performed as the groups were defined based on clinical diagnosis.
Blinding	Blinding was not used. As stated in the paper: To ensure a streamlined experience for the patients, the first four scanning sessions were controls only, the next four sessions included participants with bipolar disorder and controls.

Reporting for specific materials, systems and methods

We require information from authors about some types of materials, experimental systems and methods used in many studies. Here, indicate whether each material, system or method listed is relevant to your study. If you are not sure if a list item applies to your research, read the appropriate section before selecting a response.

Materials & experimental systems

n/a	Involved in the study
☒	☐ Antibodies
☒	☐ Eukaryotic cell lines
☒	☐ Palaeontology and archaeology
☒	☐ Animals and other organisms
☒	☐ Clinical data
☒	☐ Dual use research of concern

Methods

n/a	Involved in the study
☒	☐ ChIP-seq
☒	☐ Flow cytometry
☐	☒ MRI-based neuroimaging

Magnetic resonance imaging

Experimental design

Design type: n/a, not fMRI

Design specifications: n/a

Behavioral performance measures: n/a

Acquisition

Imaging type(s): Structural: T1-weighted; T1-weighted to calculate T1-relaxation, Diffusion-tensor. Pseudo-Continuous Arterial Spin Labeling for cerebral blood flow.

Field strength: 3 Tesla

Sequence & imaging parameters: Images were acquired on a GE Discovery MR750 (General Electric Medical Systems, Milwaukee, WI, Spftware version DV26.0_R02_1810.b. Each MRI session included the following acquisitions: T1-weighted imaging (3D BRAVO, sagittal slices, 0.9 mm3 voxels, echo time: 3.02ms, repetition time: 6.77ms, flip angle: 8°), 3D pseudo-continuous arterial spin labeling imaging (pCASL, 3.0mm3 voxels, axial slices, echo time: 11.11ms, repetition time: 5050ms, flip angle: 111°, post-label delay: 2025ms), diffusion-weighted imaging (b-value=1000, 2mm3 voxels, 32 diffusion directions; posterior to anterior encoding direction; four b-value=0, repetition time ~7142ms). An 8-volume non-diffusion weighted sequence was also acquired with the same parameters, but with the encoding direction anterior to posterior for B0-induced distortion correction. Four acquisitions were used to calculate calibrated quantitative T1 relaxation time maps with B1 correction37 (sagittal slices, two high resolution (1mm3) fast spoiled gradient echo (fSPGR) scans with whole-brain excitation, echo times: 4.4ms; repetition times 10.6ms; and flip angles of 3°(Flip3) and 14° (Flip14); two lower resolution (4mm3) SPGR scans with repetition times 50-60ms, echo time 5ms, flip angles 130° and 150°).

Area of acquisition: whole brain

Diffusion MRI: ☒ Used ☐ Not used

Parameters: see above, cardiac gating was not used

Preprocessing

Preprocessing software:
· Freesurfer (version 7.1.1, http://surfer.nmr.mgh.harvard.edu/fswiki)
· Advanced Normalization Tools (ANTs) version 2.3.3 (http://stnava.github.io/ANTs).
· N4 bias correction was used as implemented in ANTs.
· From ANTs: antsBrainExtraction.sh with the OASIS-30 Atropos template41 (https://mindboggle.info/data.html).
· Human Connectome Project Multi-Modal Parcellation atlas (version 1, HCP_MMP1, MMP1.0 210V, https://balsa.wustl.edu/976l8) via multiAltasTT (https://github.com/faskowit/multiAtlasTT.git)
· FSL (version 6.0.3, https://fsl.fmrib.ox.ac.uk/fsl/fslwiki/FSL)
· Johns Hopkins University white matter atlases provided with the FSL software (https://fsl.fmrib.ox.ac.uk/fsl/fslwiki/Atlases).
· A modified version of FSL's registration procedures implemented ANTs linear and non-linear transformations prior to white matter skeletonization (build e934eb2, https://github.com/trislett/ants_tbss).

Normalization: T1-weighted: linear and nonlinear registration to unbiased template using Freesurfer.

Diffusion weighted: Within subject averaged non-DW templates were created. ANTs linear and nonlinear transformations used for spatial normalization.
CBF: linear registration of difference images to non-DW template, and then linear & nonlinear registrtation to T1w
T1 relaxation: linear registration to 'half-way' space between flip3 and flip 14 images; linear and nonlinear registration of T1 relaxation maps to unbiased T1w, and MNI152

Normalization template: MNI152

Noise and artifact removal: n/a, not fMRI

Volume censoring: n/a, not fMRI

Statistical modeling & inference

Model type and settings: For ROI-wise analyses only: Mass-multivariate analysis of group-level and mass-univariate analysis of subject-level effects. Subject-level effects were sinusoid oscillation regression coefficients. Group-level effects were intercept only.

Effect(s) tested: We estimated the 24-hr oscillation amplitude/acrophase from each subject's 8-9 data points and tested the 8-24 subjects for a non-zero group-level amplitude with an F-test.

Specify type of analysis: ☐ Whole brain ☐ ROI-based ☒ Both

Anatomical location(s): Grey matter: ROIs from Freesurfer (v7.1.1, subcortical); HCP_MMP1 (cortical); White matter: JHU ICBM DTI-81; JHU_ICBM as provided by FSL

Statistic type for inference (See Eklund et al. 2016): n/a, not fMRI

Correction: For ROI analyses, False Discovery Rate (FDR) was applied to each MRI metric

Models & analysis

n/a	Involved in the study
☒	☐ Functional and/or effective connectivity
☒	☐ Graph analysis
☐	☒ Multivariate modeling or predictive analysis

Multivariate modeling and predictive analysis: Group-level oscillations were tested with a multivariate F-test on the bivariate outcomes obtained from each subject.
